# Pericardial effusion as the only manifestation of infection with *Francisella tularensis*: a case report

**DOI:** 10.1186/1752-1947-2-206

**Published:** 2008-06-13

**Authors:** Cécile Landais, Pierre-Yves Levy, Gilbert Habib, Didier Raoult

**Affiliations:** 1 Université de la Méditerranée, Unité des Rickettsies, CNRS UMR 6236 IRD 3R198, IFR 48, Faculté de Médecine, Boulevard Jean Moulin, 13385 Marseille cedex 05, France; 2 Department of Cardiology, Timone Hospital, Marseille, France

## Abstract

**Introduction:**

*Francisella tularensis*, a facultative intracellular Gram-negative bacterium, has rarely been reported as an agent of pericarditis, generally described as a complication of tularemia sepsis. *F. tularensis *is a fastidious organism that grows poorly on standard culture media and diagnosis is usually based on serological tests. However, cross-reactions may occur. Western blotting allows the correct diagnosis.

**Case presentation:**

A non-smoking 53-year-old woman was admitted to hospital with a large posterior pericardial effusion. Serological tests showed a seroconversion in antibody titers to *F. tularensis *(IgG titer = 400) and *Legionella pneumophila *(IgG titer = 512). *F. tularensis *was identified by Western immunoblotting following cross-adsorption. The patient reported close contact with rabbits 2 weeks prior to the beginning of symptoms of pericarditis.

**Conclusion:**

We report a rare case of pericardial effusion as the only manifestation of infection by *F. tularensis*. The etiological diagnosis is based on serology. Western blotting and cross-adsorption allow differential diagnosis.

## Introduction

Tularemia, caused by the facultative intracellular Gram-negative bacterium *Francisella tularensis*, is endemic in certain areas of the northern hemisphere. In France, it is a rare disease, being diagnosed mainly in the north-eastern part of the country. More than 250 animal species can be infected by *F. tularensis*. Small rodents are the main natural hosts (reservoir), and blood-sucking ectoparasites are the most important vectors. In addition, the bacteria are quite stable in the environment under humid and cold conditions. Humans can acquire the infection through the bites of infected arthropods or after contact with infected animals or contaminated water, food, dust and aerosols. *F. tularensis *comprises two predominant subspecies: *F. tularensis *spp. *tularensis *(biovar type A) and *F. tularensis *spp. *holarctica *(biovar type B), which is the most commonly encountered in Europe but which is less virulent and non-lethal in humans [1]. In areas of high endemicity, physicians are aware of the six classic forms of tularemia: ulceroglandular, glandular, oculoglandular, pharyngeal, typhoidal and pneumonic [2]. Although non-lethal, *F. tularensis *spp. *holarctica *(biovar type B) may cause severe disease, and in the case of delay of appropriate therapy, the course may be long-lasting and complicated.

*F. tularensis *has rarely been reported, to date, as an agent of pericarditis. We report a case of pericardial effusion due to this pathogen.

## Case presentation

A non-smoking 53-year-old woman on vacation in the French Alps was admitted to a hospital in July 2005 because of sudden and severe dyspnea at rest and chest pain. These symptoms were improved by anteflexion. She also had a one week history of fever (39°C), asthenia and abdominal pain. An electrocardiogram showed depression of the PR segment, moderate sinus tachycardia and diffuse ST segment elevation, which was concave upwards, was present in the anterior leads. A transthoracic echocardiograph revealed a large posterior pericardial effusion. A chest x-ray and a computed tomography scan showed cardiac enlargement, pleural effusion and interstitial pneumonia. A urine test for *Legionella pneumophila *1 was negative. Serological tests for *Coxiella burnetii*, *Bartonella *spp., *Chlamydia *spp.*, L. pneumophila, Brucella *spp., *Mycoplasma pneumoniae*, *Borrelia burgdorferi*, *Toxoplasma gondii*, cytomegalovirus, human immunodeficiency virus, hepatitis C and enterovirus were performed and were all negative. The patient's serum C-reactive protein level and erythrocyte sedimentation rate (first hour) were high at 186 mg/liter and 130 mm/hour, respectively, and her white blood cell count was 12 g/liter. Empirical treatment with amoxicillin, 6 g per day, and ofloxacin, 10 mg/kg per day, was initiated. The fever resolved completely within 2 weeks and the volume of pericardial fluid decreased significantly.

**Figure 1:**
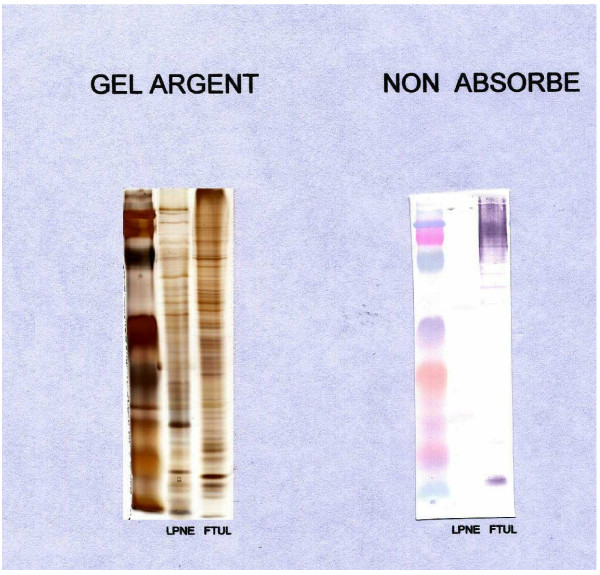
**Western immunoblotting**. *Legionella pneumophilia *(LPNE) and *Francisella tularensis *(FTUL).

Serological tests, performed on a second serum sample 2 months later during a consultation at the Department of Clinical Microbiology in Marseilles, showed a seroconversion in antibody titers to *F. tularensis *(IgG titer = 400) and *L. pneumophila *(IgG titer = 512). *F. tularensis *was identified by Western immunoblotting following cross-adsorption (Figure 1). The patient retrospectively reported close contact with rabbits 2 weeks prior to the beginning of the symptoms of pericarditis.

## Discussion

To study the etiological diagnosis of pericardial effusion, we previously developed a diagnostic strategy that recommends the systematic use of a combination of non-invasive tests used to diagnose benign pericardial effusions [3]. This strategy leads to a reduction in the number of pericarditis cases classified as idiopathic compared with an intuitive prescription of tests [4,5]. In our previous experience of the etiological diagnosis of 204 cases of pericardial effusions [3], *F. tularensis *was never found. Rare cardiac complications have been reported in tularemic infections including one case of endocarditis [6]. In 1958, a historic description reported 28 cases of pericarditis due to tularemia [7]. The postulate at that time was that pericarditis developed by direct extension from adjacent pleural effusion or from areas of pneumonia. Rare cases of pericarditis have been described as complications of tularemia sepsis caused by hematogenic spread during the course of disease [2]. In our case, the pericardial effusion was the only clinical manifestation of the disease.

Diagnosis is guided by clinical symptoms and confirmed by serological results or culture. *F. tularensis *is a fastidious organism that grows poorly on standard culture media. Owing to achievements in technology, however, tularemia can now be rapidly and specifically diagnosed. Conventional polymerase chain reaction has been successfully applied on wound specimens of patients acquiring tularemia, and prospects for application on other specimens in humans are promising [8].

Serological testing, especially the indirect immunofluorescent antibody assay, remains the most commonly used diagnostic test and is frequently the only available means for the laboratory diagnosis of *F. tularensis*. Several serology methods are available, including tube agglutination, microagglutination, hemagglutination and enzyme-linked immunosorbent assays [1]. Serological diagnosis requires a four-fold or greater rise in antibody titer between acute-phase and convalescent-phase sera. IgM, IgA and IgG antibodies appear simultaneously after initial infection and IgM antibodies can last for many years [9]. Initially, Evans reported that *Brucella *spp. and *F. tularensis *contained common antigens [2]. Some serological cross-reactions have been described, especially in IgM with *Brucella *spp., *Proteus *OX19, and *Yersinia pestis *[10]. Serological cross-reactions have also been encountered between *Legionella *and *Campylobacter*, *Mycoplasma*, *Chlamydia*, *Citrobacter freundii*, *Leptospira*, and some *mycobacteria *[11]. To the best of the authors' knowledge, there is no previous description of serological cross-reaction between *F. tularensis *and *L. pneumophila*. Western immunoblotting may be useful in making etiological diagnoses and overcoming confusing cross-reactivity. In our case, the specific antibodies reactive to *F. tularensis *were detectable (FTUL, Figure 1).

## Conclusion

Pericardial effusion due to *F. tularensis *is a rare complication. Serological cross-reactivity between *Francisella *and other bacteria precludes identification of the species causing the infection when using migration inhibitory factor. However, Western immunoblotting may help to overcome some of these limitations in situations where sera are the only available samples.

## Competing interests

The authors declare that they have no competing interests.

## Authors' contributions

CL participated in the analysis of bacterial tests and in writing a first draft, PYL participated in collecting the data and in following the patient's case, and contributed to the discussion, GH participated in the diagnosis of pericardial effusion in Marseille and generated the data, DR participated in the generation of the data, provided the results of the bacterial tests and contributed to the discussion. All authors read and approved the final manuscript.

## Consent

Written informed consent was obtained from the patient for publication of this case report and any accompanying images. A copy of the written consent is available for review by the Editor-in-Chief of this journal.
